# Antidepressant treatment response is modulated by genetic and environmental factors and their interactions

**DOI:** 10.1186/1744-859X-13-17

**Published:** 2014-06-13

**Authors:** Dávid Kovacs, Xénia Gonda, Péter Petschner, Andrea Edes, Nóra Eszlari, György Bagdy, Gabriella Juhasz

**Affiliations:** 1 Department of Pharmacodynamics, Faculty of Pharmacy, Semmelweis University, 1089 Budapest, Hungary; 2 MTA-SE Neuropsychopharmacology and Neurochemistry Research Group, 1089 Budapest, Hungary; 3 Department of Clinical and Theoretical Mental Health, Kutvolgyi Clinical Center, Semmelweis University, 1125 Budapest, Hungary; 4 Neuroscience and Psychiatry Unit, School of Community Based Medicine, Faculty of Medical and Human Sciences, The University of Manchester, UK and Manchester Academic Health Sciences Centre, M13 9PT Manchester, UK

## Abstract

Although there is a wide variety of antidepressants with different mechanisms of action available, the efficacy of treatment is not satisfactory. Genetic factors are presumed to play a role in differences in medication response; however, available evidence is controversial. Even genome-wide association studies failed to identify genes or regions which would consequently influence treatment response. We conducted a literature review in order to uncover possible mechanisms concealing the direct effects of genetic variants, focusing mainly on reports from large-scale studies including STAR*D or GENDEP. We observed that inclusion of environmental factors, gene-environment and gene-gene interactions in the model improves the probability of identifying genetic modulator effects of antidepressant response. It could be difficult to determine which allele of a polymorphism is the risk factor for poor treatment outcome because depending on the acting environmental factors different alleles could be advantageous to improve treatment response. Moreover, genetic variants tend to show better association with certain intermediate phenotypes linked to depression because these are more objective and detectable than traditional treatment outcomes. Thus, detailed modeling of environmental factors and their interactions with different genetic pathways could significantly improve our understanding of antidepressant efficacy. In addition, the complexity of depression itself demands a more comprehensive analysis of symptom trajectories if we are to extract useful information which could be used in the personalization of antidepressant treatment.

## Introduction

Major depressive disorder (MDD) is a great burden for our society owing to its high prevalence rate and disabling symptoms [1]. Effective medications against MDD show significantly better efficacy compared to placebo, especially in severe cases [2]. However, less than half of the patients achieve remission with the first prescribed antidepressant [3]. Family studies indicate that variability of antidepressant response shows a high heritability; therefore, just as in the case of the development of MDD, the role of genetic factors can be assumed [4]. However, there are no consequently replicable findings about common polymorphisms associated with antidepressant response [5].

One possible explanation is that antidepressant response is a multifactorial and polygenic phenotype determined by several common variants, and each individual SNP is only responsible for a small fraction of heritability which makes them invisible in statistical analyses with a reliable confidence rate. Nonetheless, rare genetic variants generally have a larger impact; so, this might give us hope to identify genetic variants with more impressing effects, although the current sample sizes of most studies are too small to detect them [6]. Furthermore, environmental factors such as childhood maltreatment and stressful life events in several studies showed independent effects on selective serotonin reuptake inhibitor (SSRI) response, and in some cases, also in significant interaction with genetic polymorphisms [7,8]. Although there are several genes showing consistent interaction with certain environmental factors in the development of depression, only a fraction of these interactions was tested in the case of antidepressant treatment (Table 1) [9]. Thus, in this review, we aimed to summarize our current knowledge about the possible genetic pathways and variants associated with antidepressant response, and potentially interacting with environmental predictors, focusing on the widely used SSRIs and their investigation in large-scale GWA-studies including STAR*D, GENDEP, and MARS.

**Table 1:** Positive results for gene-environment interactions behind antidepressant treatment response

**Gene variant**	**Interacting factor**	**Outcome**	**Sample size**	**References**	**Measuring methods**
5-HTTLPR	Stressful life events	Escitalopram response	674	[10]	Montgomery-Asberg Depression Rating Scale (MADRS),
					List of Threatening Experiences questionnaire(LTE-Q)
5-HTTLPR	Adverse life events	Fluvoxamine response	159	[11]	Hamilton Rating Scale for Depression (HAMD)
					Life Events and Difficulty Schedule(LEDS)
SLC6A2 polymorphism	Childhood abuse	Antidepressant treatment response	308	[12]	Hamilton Depression Rating Scale (HDRS-17)
					Childhood Trauma Questionnaire(28 item Short Form, CTQ-SF)
5-HTR1B, TPH2 polymorphisms	5-HTR1B with negative life events, TPH2 with childhood trauma	Antidepressant treatment response	308	[13]	Hamilton Depression Rating Scale (HDRS-17)
					Childhood Trauma Questionnaire(28-item Short Form, CTQ-SF)
					Life Events Scale (LES)

## Methods

We searched PubMed using search terms ‘antidepressant treatment response’ or ‘SSRI treatment response’ AND ‘gene x environment’ or ‘genetic’ or ‘GWAS’. We included papers on human subjects published in English language before 2014 February. Next, the retrieved abstracts were investigated for quality of evidence. We also included papers from the reference lists of included studies. The main focus of the present review was large-scale GWA-studies including STAR*D, GENDEP, and MARS. Regarding candidate gene studies, detailed reviews are available [9,14]; therefore, we only discussed genes that are likely to show gene-by-environment interactions (G × E). Namely, the serotonin transporter gene (*SLC6A4*), as the gene encoding the main target of SSRIs, and the most investigated genes involved in neuroplasticity/neurodevelopment and inflammation that also have major roles in antidepressant response based on animal studies [15]. Thus, the present review was not intended to exhaustively summarize the literature.

### Candidate gene studies

#### ***Serotonergic system: 5-HTTLPR***

Lesch et al. in 1996described a *SLC6A4* gene-linked polymorphic region (*5-HTTLPR*) upstream of the *5-HTT* coding sequence *(SLC6A4),* having a short and long variant which robustly influences transcriptional activity. In this study, carriers of two long alleles synthesized 1.4 to 1.7 times more mRNA compared to those who carried the short variant at least in one copy [16]. Lesch et al. have found an association between 5-HTTLPR and the neuroticism personality trait as measured by NEO-PI-R, which has been identified as a risk factor in the development of MDD. Subsequently, several studies attempted to clarify the link between MDD and *5-HTTLPR*, with contradictory results. One of the most recent meta-analyses concluded that it has a small but significant effect; however, the authors warn about publication bias that might interfere with these findings [17].

The main site of action of SSRIs is the 5-HTT protein, so extensive research started in order to find the link between *5-HTTLPR* and SSRI response. Studies in mixed-race samples yielded contradictory findings, however, in Caucasian patient samples there is suggestive evidence pointing to better SSRI response associated with presence of the long allele [18].

Besides the independent effects of *5-HTTLPR*, its interactions with environmental factors (G × E interactions) were also reported. Short allele carriers who also experienced childhood maltreatment and recent life events showed increased vulnerability to stress, increased severity of depression, and reported persistent MDD rather than single, time-limited episodes [7,8,19]. Depression severity is a well-known risk factor of unfavorable treatment response [6], and childhood maltreatment is one of the main environmental predictors of severe depression [20]. Thus, it can be assumed that childhood maltreatment in interaction with the *5-HTTLPR* can influence SSRI efficacy, although it has not been tested as yet. However, 5-HTTLPR significantly interacted with recent stressful life events on escitalopram efficacy [10]. This interaction suggests that genetic polymorphisms alone have rather poor predicting capabilities but with their specific environmental interactions, a more reliable predictor complex can be formed.

Besides the *5-HTTLPR*, further polymorphisms within the *SLC6A4* gene were identified and in some cases, found to be associated with SSRI response. For example, in a large-scale study, Stin4 showed similar interaction with stressful life events and modified SSRI response independent of *5-HTTLPR*[10]. Furthermore, an SNP has been identified within the long allele of *5-HTTLPR* which could abolish the greater expression rate of the long variant, making it functionally equal to the short variant [21]. Therefore, we can conclude that *5-HTTLPR* has important interactions with other genetic variants within the serotonin transporter gene *(SLC6A4)* and possibly with other genes and environmental stressors, so examining its effect independently from all other factors seems to be an ineffective approach. Rather, focusing on the interactions of *5-HTTLPR* already produced valuable information concerning SSRI response, and could possibly aid biomarker research for personalized treatment in the future.

#### ***Neuroplasticity: BDNF***

Brain-derived neurotrophic factor (*BDNF*) is the most common neurotrophin in the human brain. It has a confirmed role in brain development and synaptic plasticity, and *via* these biological processes, it modulates personality development and cognitive functions [22]. According to early animal studies*, BDNF* showed promising features to be a probable biomarker for depression. *BDNF* infusion to the midbrain reduced pain sensitivity and learned helplessness in rat models of depression [23], while other reports found elevated BDNF levels in SSRI-treated animals [24].

Indeed, physiological relationships have been identified between 5-HT and BDNF in neurogenesis and neuroplasticity. They activate intracellular cascades leading to the activation of the same set of transcriptional factors, (e.g. cAMP response element*-*binding protein(CREB),CREB-binding protein (CBP), forkhead box protein O (FOXO), nerve growth factor-inducible factor A (NGFI-A)) which can prevent cell death or induce neuronal growth [25]. Experimental evidence is available about the facilitating effect of SSRIs on cognitive functions, spatial memory, hippocampal neurogenesis, and brain recovery after injury possibly through increased BDNF production [25].

A common SNP (*rs6265*) in the coding exon of the *BDNF* gene leading to a valin (Val) to methionin (Met) change has been identified, and examined comprehensively with the minor, Met coding variant showing reduced activity and contributing to the development of MDD, especially in elderly patients; while the higher activity Val allele was associated with bipolar depression, and substance abuse according to a recent meta-analysis [22]. The Val66Met polymorphism alters the regulated release of BDNF, while the constitutive release remains intact. In addition, Met66 carriers often exhibit poorer episodic memory, and abnormal hippocampal activity [26].

Many studies investigating the efficacy of SSRIs yielded inconsistent results about the Val66Met polymorphism, and the hypothesis of Val66 variant facilitating better response is not confirmed [14,27]. Some reports even found the Met66 allele associated with better response to SSRIs [28]. However, it is important to note that in animal models, overexpression of *BDNF* in the hippocampus causes antidepressant-like effect while in the ventral tegmental area or nucleus accumbens, it is associated with depression-like behavior [29,30]. These results suggest that proper *BDNF* function which is a crucial mediator of activity-dependent neuronal plasticity is important for a favorable SSRI response. In addition, other factors should be taken into consideration in the case of *BDNF*’s effect on SSRI efficacy.

A recent meta-analysis found a significant interaction between stressful life events and *BDNF* Val66Met polymorphism. Met66 carriers showed a higher probability to develop depression if stressful life events were present [31]. Another study reported a more complex interaction, where the Met66 variant with other SNPs in the *BDNF-TRkB-CREB1* pathway showed interaction with childhood adversity and increased the risk for depression, while the Val66 variant among other SNPs was associated with a maladaptive cognitive response style for life stress, called rumination, which is also a risk factor for depression [32]. It seems likely that both alleles of the Val66Met polymorphism of *BDNF* have their own spectrum of vulnerability by modulating consequences of specific environmental factors, and therefore, they cannot be generalized as either favorable or unfavorable alleles. Moreover, it is important to note that introducing intermediate phenotypes, like rumination, hippocampal volume or stress hormone response, can uncover novel mechanisms that are responsible for contradictory findings in gene-main effect studies. Intermediate phenotypes are genetically determined traits that in neuropsychiatry represent neurobiological processes with a causal role in the disease pathway [33,34]. Considering these, a comprehensive analysis of *BDNF* G × E interaction effects on SSRI response and using intermediate phenotypes of antidepressant response could explain more variability than previous studies focusing on the independent effects of the SNPs and only one outcome measure. Unfortunately, there is no such study available yet, partially due to the difficulty of reliably measuring complex environmental factors.

Another important confounding factor is the dependence of *BDNF* gene expression on an enormous number of other genes, and their polymorphisms in the neuroplasticity pathway. For example, in rat models, SSRI treatment could not increase *BDNF* levels and exert its behavioral effect in the absence of the *CREB* gene [25]. Nonetheless, a large-scale mRNA expression study which measured changes in SSRI-treated mice found that the signaling pathway including *BDNF-TrkB-CREB1* showed the best association with SSRI treatment [35]. Thus, searching for SNPs associated with the *BDNF* pathway in interaction with environmental factors seems now a fruitful approach for candidate gene studies to produce useable results.

#### ***Inflammation***

Despite the fact that the monoaminergic theory is the currently accepted hypothesis of MDD, there is undeniable evidence about the existence of several other factors playing the role in the background of depression including inflammation-associated processes. In the wide comorbidity spectrum of MDD, there are many disorders associated with strong underlying inflammatory mechanisms [36]. Moreover, in MDD patients compared to controls a significantly higher concentration of proinflammatory cytokines, such as TNF-α, IL-1β, and IL-6 can be measured [37]. In addition, administration of TNF-α antagonist etanercept to patients with psoriasis was reported to reduce MDD symptoms. In contrast, IFN-α and IL-2 treatment induces depressive symptoms as a common side effect [38]. Based on these observations, we can conclude that the inflammation pathway has a significant effect in depression. However, antidepressant treatment also has an effect on inflammatory processes, namely they lowered the levels of IL-1β, and specifically SSRI treatment reduced the IL-6 levels in MDD patients in a recent meta-analysis [39].

Another recently proposed possible biochemical link between 5-HT and inflammation may also explain the relationship between depression and inflammation. 5-HT synthesis from tryptophan can be altered by proinflammatory cytokines, through the increased activation of indolamine-2,3-dioxygenase (IDO). IDO activation promotes the production of kynurenine from tryptophan instead of 5-HT, and kynurenine has two NMDA agonist metabolites with neurotoxic properties [40]. There is experimental evidence that polymorphisms in the *IDO1* and *IDO2* genes significantly modulated citalopram response possibly by decreasing the availability of neurotoxic kynurenine pathway products [41,42].

There are several possible mechanisms proposed besides the effect on tryptophan bioavailability and neurotoxic kynurenine metabolites which can cause inflammation-associated MDD. Adaptive immune functions are impaired in MDD according to several reports, so the balance between proinflammatory and anti-inflammatory cytokines is disturbed. The prolonged pro-inflammatory effects cause increased glucocorticoid production and increased hypothalamic-pituitary-adrenal (HPA) axis activity. These consequently damage hippocampal neurons, and contribute to the development of MDD. Pro-inflammatory cytokines also disturb neurogenesis and synaptic plasticity through microglial activation, which negatively influence the survival rate of new hippocampal neurons [43].

Considering G × E interactions, a polymorphism (rs1360780) in the FKBP5 gene which codes a protein with a major role in HPA axis regulation interacts with childhood maltreatment on depression severity and development [44]. Although this gene influenced antidepressant response in the STAR*D study, there is no data published whether it is modulated by life stresses [45]. Glucocorticoid receptor gene *(NR3C1*) polymorphisms were also found to be predictors of response to both tricyclic and SSRI antidepressants on a large sample of MDD patients [27]. These findings suggest that preventing production of proinflammatory cytokines can possibly increase efficacy of antidepressants. For example, celecoxib combined with SSRIs showed significantly better efficacy than placebo plus SSRI [43], although some contradictory findings were also published [46,47].

In summary, SSRIs down-regulate proinflammatory processes while anti-inflammatory treatment may enhance SSRI efficacy. Nevertheless, the role of inflammation on the outcome of antidepressant treatment is usually neglected, despite the fact that several well-established lines of evidence suggest its importance. Taking biomarkers of inflammation into account in genetic association studies on antidepressant treatment, similarly to other environmental effects such as childhood maltreatment or stressful life events, may reveal several new aspects of SSRI efficacy. However, there is no such study published as yet to the best of our knowledge. A possible difficulty of these studies is that ‘classic’ environmental factors such as stressful life events already include conditions which have a unique and well-described relationship with inflammation, like different medical illnesses. Therefore, a more precise measurement of environmental effects is required to improve the future G × E interaction studies considering inflammation-associated gene regions.

#### ***Other potential mechanisms***

There are many candidate gene studies which provided promising results about genes and polymorphisms related to antidepressant response, for example in cannabinoid, glutamate, dopamine, or norepinephrine pathways [48,49]. Comprehensive discussion of these pathways is beyond the scope of the present paper which focuses on G × E interactions. Furthermore, we only aimed to represent findings of antidepressant treatment response, not depression as a disorder, on which a thorough discussion can be found in other recent papers, e.g., by Mandelli and Serretti [9].

### GWAS

Genome-wide association studies (GWAS) use entirely different methods than previously presented candidate gene studies. GWAS do not rely on a set of genes expected to predict the outcome. The inclusion criterion is merely the minor allele frequency (MAF), which describes the occurrence rate of the polymorphism. Therefore, GWAS scan hundred thousands of SNPs in an appropriately large sample of patients. The high volume of genotyped samples give immense statistical power to GWAS studies, however, way more rigorous significance levels (*p* ≤ 5 × 10^-8^) have to be employed in order to avoid false-positive results. In the following three sections, we would like to review the three biggest GWAS studies on antidepressant efficacy.

#### ***GENDEP***

The Genome-based Therapeutic Drugs for Depression (GENDEP) study examined 706 MDD patients treated with nortriptyline or escitalopram for 12 weeks. On a genome-wide significance level, the gene coding uronyl-2-sulphotransferase (*UST*) was associated with nortriptyline response. On a suggestive level, *IL-11* expression was associated with escitalopram response, and two intergenic regions on chromosome 1 and 10 were found to be linked with response to both medications. On a lower level of significance, *IL-6* appeared as a noticeable modulator, which further supports the significance of the role of the proinflammatory pathway in SSRI efficacy and the inflammation theory of depression. The other identified predictor regions were pretty unexpected. *UST* is an enzyme necessary to produce oversulfated proteoglycans, influencing neurogenesis, and neuronal migration. The intergenic polymorphism found on chromosome 1 is close to the coding sequence expressing zinc finger protein 326 (*ZNF326*). On chromosome 10, the closest gene is the plexin domain containing 2 (*PLXDC2*). They are both involved in the structural changes of chromatin, but the exact mechanism on SSRI effect is unknown. However, these findings suggest that polymorphisms in non-coding areas which possibly influence the transcription rate of other gene-coding regions have the same or even more power to modulate SSRI efficacy [50].

Besides genetic factors, age, depression severity, and stressful life events showed significant association with SSRI response. Younger patients with the lowest baseline depression severity showed better response to escitalopram. Interestingly, subjects with one or more stressful life events also responded better to escitalopram, suggesting the role of the serotonergic system in the development of life stress-induced depression [10].

#### STAR*D

In the Sequenced Treatment Alternatives to Relieve Depression (STAR*D) study, all 1,493 patients were treated initially with citalopram in a naturalistic design, with no placebo control or serum level assessments to filter out no real medication effect and non-compliance [51]. There was no genome-wide significant association found among the 430,198 SNPs tested. The top two associated polymorphisms were in non-coding areas, one is between the ubiquitin protein ligase E3C gene (*UBE3C*) and motor neuron and pancreas homebox 1 (*MNX1 or HLXB9*), and the other is near the bone morphogenic protein 7 *(BMP7*) gene. *UBE3C* signals proteins for degradation, and *MNX1 *has a role in embryonic brain development. Just like in GENDEP, the top findings are in non-coding regions, close to non-expected genes [52]. Comparing the genetic properties of sustained responder groups to those of non-sustained responders, the top 25 SNPs were again intronic or intergenic. Although there is a lower chance of placebo effect or non-compliance in sustained responder than in non-sustained responders, the STAR*D top findings were non-replicable in the GENDEP sample [53].

Apart from genetic features, minority status, socioeconomic disadvantage, comorbid conditions, lower functioning and quality of life, and anxious or melancholic features were found to be unfavorable on the outcome [54]. Among the sociodemographic predictors, simultaneous presence of higher education, higher income, not living alone, and good employment status were highly correlated with remission [55].

#### ***MARS***

The Munich Antidepressant Response Signature (MARS) included 339 patients, 88.8% with MDD and 11.2% with BPD, all of whom were Caucasian, and 85.1% from German descent [56].The *CDH17* gene polymorphism rs6989467 showed the most significant association with treatment outcome. The genome-wide testing was also accomplished on an independent German sample (*n* = 361), and an intergenic SNP between *AK090788* and *PDE10A* genes showed the strongest association. None of these findings could achieve genome-wide significance, and the authors concluded that these genes have a rather modest effect on SSRI response. Suspecting multiple genes, pathway analysis was performed, which identified three gene clusters. The first cluster is centered on fibronectin 1 (FN1), including transcriptional factors and the substrate, and receptor genes of ephrin-A5, which has a role in late-stage nervous system development, and is associated with hippocampal neural plasticity. The second cluster has risk genes for cardiovascular and metabolic disorders, which show a high comorbidity with depression, and the third is related to GABA and glutamatergic neurotransmission [56]. Signaling pathway analysis is a progressive approach to identify genes with cumulating effects on complex phenotypes like depression, and could be a very fruitful way to search for potential target molecules in the future.

In the same sample, HPA axis hyperactivity was tested by the combined dexamethasone suppression/CRH stimulation (DEX-CRH) test. As proposed earlier, HPA hyperactivity most likely caused by impaired negative feedback control in MDD patients was associated with depression severity. After 5 weeks of antidepressant treatment, normalization in HPA hyperactivity was observed in remitters, but not in non-remitters. A single DEX-CRH in males and repeated tests in females were able to predict antidepressant treatment outcome [57]. Therefore, testing HPA axis function, as a biomarker of the body response to environmental stressors, seems to be an interesting approach to predict SSRI efficacy prior to treatment.

### Meta-analysis

The three studies above were summarized by a meta-analysis, which could not find a genome-wide significant association in case of any of the 1.2 million analyzed polymorphisms [5]. Although the authors concluded that there are no reliable predictor gene regions or a set of common SNPs, valuable conclusions could be made about the relevance and capabilities of GWAS studies. Methodological problems such as definition of responders/non-responders, or choosing the inclusion criteria, and determining the study design can cause more interference with the results if they are expected to meet the GWAS level of significance [51]. Individual differences in symptom trajectories were found to be important predictors of treatment outcome itself further supporting that MDD is a heterogeneous disorder. For example, patients with less activity and lack of interest showed worse response to antidepressant treatment [58]. In contrast, cognitive symptoms were found to be more responsive to SSRI treatment [59]. Based on this experience, the more accurate declaration of our expectations on SSRI response should help find more significant associations. Moreover, integrating measures of non-subjective intermediate phenotypes in the symptom trajectory seems to be a promising approach. For example, neuroimaging-based intermediate phenotypes can help us better understand the brain processes behind symptom trajectories [60]. Although it is not possible to scan enough patients with neuroimaging techniques for a GWA study, these results can help us refine our phenotype-measuring system and develop more biology-related categories to work with.

Another crippling deficiency of the GWAS approach is ignoring environmental factors, while in many cases, they show better association with the response than genetic polymorphisms [55]. In the future, new more powerful statistical methods, like the Bayesian network analysis, must be developed in order to take environmental factors into consideration [61,62].

## Limitations of genetic studies on antidepressant response

The limitation of G × E interaction studies in psychiatric disorders was discussed comprehensively in a recent review by Uher [7]. Therefore, here we only highlight some major points. One important problem is the heterogeneity of the measured phenotypes. We mentioned above that the inconsistent definition of responders or non-responders, the lack of detailed investigation of symptom trajectories (e.g.: anhedonia versus cognitive symptoms) and antidepressant response-related intermediate phenotypes all hinder the identification of genetic risk factors. In addition, recall bias and the use of different life stressor measures confounds present findings related to environmental factors. A more detailed and precise measure of life stresses is required because certain genes only shape the vulnerability profile toward specific life events, e.g., early versus late life events seem to have different interaction patterns with certain genes and polymorphisms. However, they could be equally important. Early life stressors exert their effects on a developing brain that facilitates more intense changes in neural circuits which can exert long-standing negative effects. However, recent negative life events frequently trigger a cascade that eventually leads to negative neuroplastic changes. Another major problem is that present statistical methods are not powerful enough to detect G × E interactions with biological consequences and cannot handle such big datasets as would be required for genome-wide G × E (GEWI) studies. Therefore the development of new statistical methods is essential for further results. An additional problem in these studies is that hidden relatedness within the population or different ethnicity may cause stratification bias. For example, there are several differences between Caucasian and Asian patients considering the genetic background of antidepressant response. Although GWAS studies control their calculations for possible stratification biases by using specific ancestry markers, these are usually not available for candidate gene studies.

## Discussion

Investigation of common polymorphisms associated with antidepressant response showed contradictory results, even in case of logical target genes like *SLC6A4*. Recent meta-analyses only found a very small effect in the case of these polymorphisms [17], and many times, other SNPs were found to change the effect of the initial SNP [21,63,64], suggesting a more complex interaction than a one-SNP-one-phenotype relationship.

While candidate gene studies could not produce replicable results, GWAS found a multitude of genes and pathways related to antidepressant response, but the majority could not meet the strict statistical significance criteria, and the few findings which could withstand multiple testing were not replicable by other GWAS. The heterogeneity of GWAS results suggests that multiple common variants are involved in the response with a cumulative effect [5]. GWAS also highlighted the importance of genes involved in brain development and hippocampal neural plasticity besides the monoaminergic pathways.

Epigenetic factors were also identified. These changes such as methylation, or acetylation are tissue-specific, and caused by the combined effect of the DNA and environmental factors. Unfortunately the tissue-specific localization makes them only examinable in post-mortem studies with a smaller sample size, but these studies reported much larger changes in the brain than polymorphisms could ever cause [65]. Despite the accumulating evidence that G × E is a major factor in antidepressant response only a handful studies are available so far (Table 1).

As we can see the multiple steps between the examined SNPs and the outcome (which is often declared subjectively), it is reasonable why there were no replicable findings about the effect of common SNPs on antidepressant response. The magnitude of epigenetic effects combined with the multiple findings about environmental interactions must change our expectations about genetic studies [66,67]. A new model of depression proposes that each person has an individual vulnerability profile towards MDD, and MDD develops through personal maladaptive processes to cope with environmental effects. According to our current understanding, there is no common polymorphism with absolute effect on MDD, they just shape the vulnerability profile with distinct effects [68,69]. For example, the 5-HTTLPR's short variant induces vulnerability to stressful life events, but short-short carrier children seem to be more responsive to cognitive behavior therapy [70].

Therefore, the next step should be to better identify outcome variables, if we want to find significant, replicable, and useful predictors to antidepressant response. Remission and even depression should be described from a biological approach. For example, intermediate phenotypes such as HPA axis hyperactivity, pro-inflammatory cytokine concentrations or hippocampal volume, are more manageable variants for a genetic research than medical symptomatology [71]. We also have to move forward in the genetic approach of this question. A multitude of studies showed that even without the effect of the environmental factors, countless interactions are present among genetic variants, and they often entirely change the effect of an individual polymorphism. Besides that, environmental effects cannot be neglected anymore. Epigenetic studies showed us the power of combining the effect of environmental factors and genetic properties, and in the future, we shall find a way to utilize it.

## Competing interests

The authors declare that they have no competing interests.

## Authors’ contributions

DK, XG, PP, and GJ contributed to the development and content of the manuscript as well as to the interpretation of the collected literature, while GB provided guidance on both tasks during the whole progress. All authors read and approved the final manuscript and were responsible for taking the decision to submit the paper for publication.
